# Supplementary material for: A Hidradenitis Suppurativa molecular disease signature derived from patient samples by high-throughput RNA sequencing and re-analysis of previously reported transcriptomic data sets
Source: PLoS One. 2023 Apr 6;18(4):e0284047. doi: 10.1371/journal.pone.0284047 (PMC10079067; doi:10.1371/journal.pone.0284047)
Supplement: Attachment — Submitted filename: COMMENTS.docx [file pone.0284047.s005.docx]

The manuscript by Johannes Freudenberg et al. describes the transcriptomic signature of skin samples from 20 patients with HS. The manuscript is well written, the RNA-Seq is appropriate, and the results and discussion are consistent with the analysis reported. However, there is a strong discrepancy within the entire manuscript (and figures) in reference to “perilesional” and “unlesional” sites used for RNA-seq. Please clarify and modify within the entire manuscript and figures.

It would have been better to compare lesional, perilesional and non-lesional sites but it is still good and satisfying work.

**Line 49**, please delete “..” before the reference 9.

**Lines 52-56**: in the context of genetic variations underlying HS susceptibility and severity, it is important to mention first the genes involved in innate and adaptive immunity (specifying it) and then the genes involved in keratinization and autoinflammation pathways. *The references are adequate*, but I suggest to also include this novel manuscript that highlight the potential causative role of AIM2 in hidradenitis suppurativa pathogenesis (in particular of syndromic HS). The manuscript is: “Moltrasio C, Cagliani R, Sironi M, Clerici M, Pontremoli C, Maronese CA, Tricarico PM, Crovella S, Marzano AV. Autoinflammation in Syndromic Hidradenitis Suppurativa: The Role of AIM2. Vaccines (Basel). 2023 Jan 11;11(1):162. doi: 10.3390/vaccines11010162”.

Genes name must be written in italics. In addition, Cx26 is encoded by GJB2. Please write “*GJB2”* gene

without “Cx26, GJB2”.

**Line 63**: Please also consider this recent and interesting work: “de Oliveira ASLE, Bloise G, Moltrasio C, Coelho A, Agrelli A, Moura R, Tricarico PM, Jamain S, Marzano AV, Crovella S, Cavalcanti Brandão LA. Transcriptome Meta-Analysis Confirms the Hidradenitis Suppurativa Pathogenic Triad: Upregulated Inflammation, Altered Epithelial Organization, and Dysregulated Metabolic Signaling. Biomolecules. 2022 Sep 25;12(10):1371. doi: 10.3390/biom12101371”.

**Line 67**: Please also consider, after “dermcidin” this recent and interesting work: “Tricarico PM, Gratton R, Dos Santos-Silva CA, de Moura RR, Ura B, Sommella E, Campiglia P, Del Vecchio C, Moltrasio C, Berti I, D'Adamo AP, Elsherbini AMA, Staudenmaier L, Chersi K, Boniotto M, Krismer B, Schittek B, Crovella S. A rare loss-of-function genetic mutation suggest a role of dermcidin deficiency in hidradenitis suppurativa pathogenesis. Front Immunol. 2022 Dec 5;13:1060547. doi: 10.3389/fimmu.2022.1060547”.

The quality of the figures is not satisfactory.
